# Awareness of Students and Dentists on Sustainability Issues, Safety of Use and Disposal of Dental Amalgam

**DOI:** 10.3390/dj11010021

**Published:** 2023-01-08

**Authors:** Andreas Spaveras, Maria Antoniadou

**Affiliations:** Dental School, National and Kapodistrian University of Athens, 11527 Athens, Greece

**Keywords:** sustainability, dental amalgam, safety of use, environmental protection, mercury

## Abstract

Among the pillars of sustainability in health care units is environmental protection. Although an EU-wide dental amalgam phase-out legislation exists, quantities of this material are still to be found in the market, dental offices or in the mouths of patients. The purpose of this study is to record the views of dentists and dental students in Greece regarding the use and safety of dental amalgam for people and the environment as well as their attitudes towards its restriction and disposal. Materials and methods: Two different questionnaires, through Google forms, were filled by each group. Descriptive statistics were used to describe the variables. The chi-square test or the chi-square test with Yates correction was used to examine potential differences per group (*p*-value = 0.05). Overall, 564 people participated in this study; 462 (81.9%) dentists (N1) and 102 (18.1%) dental students (N2). Results: Both groups agreed that they no longer use dental amalgam often. Dentists (39.8%) and students (36.4%) consider amalgam to have a moderate burden on the environment. This answer differed significantly per year of profession and year of undergraduate studies, respectively, with dentists from 6–25 years in the profession and 4th-year students, being the least aware on the environmental footprint of dental amalgam. Further, professionals (70%) and students (60%) believe that dental amalgam has a hazardous impact on patient’s health, at all or to a small extent. For staff health, dentists reported at a moderate degree dangerous impact (32.9%) while students (36.4%), respectively. The impact on patients and staff health, were found to differ significantly per region of practicing dentistry for both groups. Finally, there were suggestions made from both groups about the necessity of information sharing on amalgam and mercury safety and the impact on the environment at the level of professional organizations. Conclusions: Students, younger dentists and those living in non-urban regions seem to be more sensitive to the environmental impact of amalgam use, disposal, and health of people. Environmental issues should be addressed thoroughly by professional organizations, enhancing relevant activities for all people involved.

## 1. Introduction

Sustainability in dentistry is an important research academic topic that has already introduced eight diverse but closely interlinked themes [1]—environmental impacts addressing CO_2_ emissions, air and water pollution, the philosophy of four Rs (reduce, reuse, recycle and rethink), policy and guidelines issues, biomedical waste management, reduced use of plastics, procurement, research and educational issues, and use of biodegradable and biomimetic materials for the oral cavity [2]. Barriers to implementation of relevant legislation issues that could excel sustainable dental enterprises, at a national or international level, are so far mainly the lack of professional and public awareness; carbon emissions arising from patient and staff transportation; challenges associated with the recovery and recycling of biomedical waste with a focus on plastics; the lack of knowledge and education into sustainable health care provision; challenges from manufacturing; and the use and disposal of dental materials such as amalgam [2,3,4,5].

Dental amalgam has served the dental community for many decades, replenishing the lost dental tissues of anterior and posterior teeth, in cavities of various sizes and classes. At the same time, opponents of dental amalgam have at times questioned its extensive use, both for health and environmental protection reasons, as well as for its inability to keep up with the aesthetic and functional needs of modern dentistry [6,7]. Negative reactions towards its use focus on a key component of the amalgam, mercury (Hg), with a b.w. ratio of approximately 50%. Hg ions, known for their toxicity, seem to be released from solidified amalgam in the form of Hg vapors. As a result, the scientific community has expressed concerns about its impact on the health of both patients with amalgam fillings in their teeth, as well as dentists and dental assistants, who are on occupational exposure to it [8,9]. At the same time, dental amalgam, from its production to its removal from the oral cavity even several years later, has been accused of polluting the environment with Hg through various routes (e.g., water and air) while it also challenges the aesthetic requirements of patients, even in posterior deciduous or permanent teeth [9,10,11]. So far, other materials and type of restorations have been suggested for minimizing the exposure of patients (especially children) and staff in Hg vapors such as composite resins, glass ionomer materials or ceramics [9,10,11,12].

Although the field of dentistry is not the main source of mercury, the United Nations World Environment Program (UNEP) Global Mercury Assessment 2013 reports that Hg from dental amalgam accounts for 21% of the world’s consumption of Hg in products, which is an accountable percentage [13]. Hg has been considered by the World Health Organization (WHO) as among the top ten chemicals that are of significant concern to public health [14]. It is released into the environment through human activities and via natural sources (e.g., volcanoes). Following its release, Hg is transported and recycled between the major environmental compartments, i.e., air, soil, and water, until it is eventually removed from the system through burial in coastal and deep-water sediments and subsurface soils [15,16]. Since it does not dissolve in the environment, Hg levels in the water supplies of the planet and the land with low vegetation are accused of health effects through the animal and plant food chain [13,14,17]. The most important route for human exposure to Hg is seafood. The chain of survival tends to accumulate higher concentrations of Hg in bigger predatory fish, like tuna or swordfish that finally enter the human chain predisposing people in greater intake of Hg. The health impacts from Hg are dose related. So far, the main concern is the impact of Hg on fetuses and young children. Hg exposure can occur in the womb due to a mother’s consumption of seafood but also due to other procedures such as the use or removal of dental amalgam fillings [18]. This can have significant and life-long impacts on the growing brain and nervous system of a baby, affecting memory, language, attention, and other skills. In Europe alone, it is estimated that more than 1.8 million children are born each year with Hg levels above recommended safe limits [19].

In 2013, the Minamata Convention on Mercury was adopted with the aim of protecting human health and the environment from the harmful effects of Hg [20]. In the field of dentistry, practical measures were taken to phase out the use of amalgam because of its content in Hg. Some countries have even banned its use. Norway was the first country to ban the use of Hg in all products in 2008, including dental amalgam, followed by Sweden and Denmark [21]. On 1 July 2018, the European Union banned the use of dental amalgam for children under 15 and for pregnant/lactating women under the scope of an EU-wide dental amalgam phase-out with the 2017/852 regulation [22]. Of course, although the Minamata Convention has been signed by 128 countries, unfortunately most of these countries have not taken practical steps in this regard [23,24]. WHO says that phasing out the use of amalgam can cause more challenges in low- and middle-income countries [2,14,21,22]. Further, in the EU, there are countries like Greece in which the use of dental amalgam is not prohibited but of course its use is diminishing. Amalgam separators, although obligatory by Greek legislation and professional organizations for new dental offices, are still not incorporated in many of the old ones.

Although the restriction of the use of amalgam as a restoration material is a modern reality, the issue of its safety while replacing old restorations, in terms of humans and the environment, is still in the center of interest and discussions, at the European and global levels. Therefore, in this paper, the knowledge, and habits of the undergraduate students of the Department of Dentistry of the National and Kapodistrian University of Athens (NKUA) and of a sample of Greek dentists regarding sustainability, safety of use and disposal of dental amalgam, were investigated. We also analyzed factors that affect its diminishing but still existing use in modern dental clinical practices.

## 2. Materials and Methods

Two different questionnaires were designed for this study, one for each group of participants (professionals or students). The questionnaires were then posted on the Google Forms platform (https://docs.google.com/forms/ (accessed on 1 October 2019) and were sent via email and social media to dentists attending the scientific events of the dental association of Attica in the Metropolitan Area of Athens (N0 = 800). The second questionnaire was designed for the undergraduate dental students of the 8th and 10th semester of their studies at the department of dentistry, National and Kapodistrian university of Athens, Greece (N1 = 260). Permission to run the relevant questionnaires was obtained by the scientific committee of the department of dentistry (2019/A18) and the dental association of Attica (license received via email on 13 October 2019).

Inclusion criteria for the first group were dentists, members of the relevant association. Exclusion criteria for the first group were non-dentists, technicians, and auxiliary staff. For the second group, participants should be: undergraduate students of the department of dentistry of the National and Kapodistrian University of Athens, studying at the 8th and 10th semester. Exclusion criteria were students of other departments of NKUA or other universities.

Both questionnaires were tested in a sample of twenty-five participants for N1 and fifteen participants of Ν2 to perceive authenticity and clarity of the questions. Anonymity of the participants was covered by the Google form platform; no personal data were collected and only one person had access to the results and analysis of the data collected. The period that the questionnaire was open was from April to October 2020.

Questionnaire A, for dentists, addressed questions about general characteristics (the time of practice of the profession, the field of practice, and the geographical area) but also questions related to their knowledge or opinions about the use of dental amalgam (source of information about amalgam, use of amalgam, aggravating environmental impact, etc.) [25]. An urban center in our research was considered a large and densely populated urban area that may include several independent administrative districts with more than 5000 people each. Questionnaire B, for students, addressed questions about the year of study, the future choice of dental field of activity and the geographical area of the country where they would practice dentistry after graduation. Other questions included enquiries about the source of information concerning amalgam and mercury impact, the frequency of use of amalgam in dental practice, aggravating environmental impact of amalgam, etc. [25,26].

Descriptive statistics were used for all categorical variables. More specifically, the frequency (percentage) was chosen as a descriptive measure. Pearson analysis was used as a graphical representation and Yates’s correction t-test analysis, depending on the percentages of the expected values in the groups. The above statistical tests were decided because all the variables of the questionnaires were categorical. In addition, the Pearson chi-square test with Yates’s correction was performed on those tests; among the categorical variables, the tables were above 2 × 2 and the cells of the expected frequencies were below 5%. A statistically significant event was considered the one with a *p* < 0.05. All statistical analyses were carried out with the statistical package IBM SPSS version 27.

## 3. Results

The response rate of the sum of participants was 564/1060 × 100 (53.2%). For group A (professional dentists), the specific rate was: 462/800 × 100 (57.75% of N1); and for group B (dental students), it was 102/260 × 100 (39.2% of N2). Both dentists (39.8%) and students (36.4%) responded that amalgam burdens the environment to a moderate extent. However, answers differed in a statistically significant way depending on the years of practice of the profession for dentists (*p* = 0.07) and year of study for students (*p* = 0.05). More specifically, dentists with 6–25 years of practice considered that amalgam has a small impact on the environment in contrast to those with ≤5 (young professionals) and >25 years (old professionals) who believed that it has a moderate effect. The 4th-year students vs. 5th-year students answered that amalgam burdens the environment at a level of “not at all” (6.3% vs. 0%), “very small” (12.5% vs. 8.4%), “small” (6.3% vs. 24.1%), “moderate” (68.8% vs. 50.6%) and “big” (6.3% vs. 16.9%), respectively.

**Figure 1:**
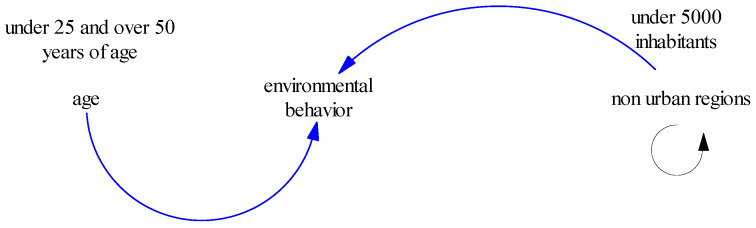
Factors affecting positively the environmental behavior of both dentists and dental students.

Furthermore, 70% of professionals answered that amalgam affects patient’s health to “not at all” or “small” extent and 60% of students to no or very small extent. However, this view was found to vary significantly per place of practice of the profession for both dentists (*p* = 0.01) and students (*p* = 0.05). More specifically, 50% of dentists practicing the profession abroad answered that amalgam fillings have a negative effect on patient’s health, while 21% of professionals practicing the profession outside urban centers and 33.9% in urban centers, gave the same answer. Regarding students, 50% of those who will practice dentistry abroad answered that amalgam fillings can have a negative effect on patient’s health to a large extent while the same answer was given by 28.6% of those who will practice the profession outside the urban center and 23.7% of those who will practice the profession in an urban center in Greece (Figure 1).

As for their opinion on the effect of amalgam on staff’s health, dentists and students answered 32.9% and 36.4% to a moderate degree, respectively. However, this view differs significantly per place of practice for professionals (*p* = 0.01) and students (*p* = 0.05). More specifically, 37.5% of dentists practicing abroad replied that amalgam fillings have no effect on staff’s health, while the same answer was given by those who practice the profession outside the urban center (18.5%) and those working in urban centers (19.7%), respectively. Additionally, regarding students, 50% of the participants who will practice dentistry abroad answered that amalgam fillings can negatively affect the health of the staff of a clinic to a small extent while the same answer was given by 7.1% of those who will practice the profession outside the urban center and 6.3% of those who will practice the profession in an urban center (Table 1 and Table 2).

Overall, most dentists seemed to never or almost never use amalgam in the restoration of posterior teeth (401 out of 462 dentists) (86.8%). A total of 50.2% dentists (232 out of 462) said that they very often try to prevent the patient, from using amalgam as a filling material if he asks for it. Concerning the process of removal of old amalgam restorations, 262 out of 462 dentists (56.7%) would use surgical suction and rubber dam isolation. Students finally stated that they were not informed about EU Regulation 2017/852 on amalgam use and disposal ways to a degree of little or not at all (63.6%, 63 out of 99).

## 4. Discussion

According to data of this study, professionals and students report that dental amalgam harms the environment but affects in a minimum degree patient’s and staff’s health. It is a material not preferred by both groups of this study. Even more, dental students in Greece no longer experience or learn the use of amalgam during their studies. Thus, this generation of young professionals will no longer be able to perform these restorations in the future anyway. The literature shows a similar diminishing trend for the use of this material. It is interesting to mention that the estimated annual demand for dental amalgam in the EU28 amounted to 27–58 t of mercury in 2018, while in 2010 it was 43% up [18]. Furthermore, it is estimated that in 2018, approximately 372 million dental restorations were carried out in the EU28. Of these, only 10% and 19% would have used dental amalgam despite differences among members of EU. Greece still counts for 5.3% of total restorations to be performed with amalgam which is the 7th highest percentage among EU country members. It is promising though that seven out of ten dentists in our study know the relevant directive EE 2017/852 and the regulation act. As far as it concerns the safety protocols about the clinical use of the material and its safe removal or disposal, it is challenging that three out of ten dentists did not even have the necessary equipment for these procedures. Of course, since the data were collected before COVID-19 pandemic, this is not representative of the safety protocols that Greek dentists were forced to encounter in everyday practice during COVID-19 pandemic and are currently using. Information shared and the safety issues that the pandemic demanded, opened the use for extended protective equipment in all clinical procedures of general dentistry. These guidelines will possibly remain active even in the post-COVID-19 era as they guarantee safety management in the dental office [27,28] thus, encountering as well, amalgam use and disposal issues.

Furthermore, our data show that direct interventions by professional associations and national dental bodies are needed to change environmental values through live experiences, education and demonstration projects that affect all generations of professionals [25,26,29]. The hazard, pollution and recyclability caused by dental amalgam can be learned by all but especially as our data show, from those dentists still working in the peak of their career (aged 28–42) [29,30,31]. The fact that only dentists before 25 and after 50 years of age were concerned about the environmental impact of dental amalgam is mostly representative of their generations. As known in the relevant literature there are generational groups which are described broadly by societal changes, economic conditions, and significant historic developments [32]. Dentists born between 1965 and 1979, aged 43 to 57 years old (generation X), represent the “forgotten generation”. They grew up during the emergence of technology into their daily lives and the seismic shifts in global affairs including environmental crisis [33]. They are better educated than the preceding generation yet less well educated than those of the Millennial generation and Generation Z [34]. Dentists born between 1980 and 1994, aged 28 to 42 years old, the Millennials, who are currently trying to build their dental career are well into the stress of an upbringing business thus they do not always have the time to organize other than their clinical engagement. Generation Z, on the contrary, with individuals born between 1995 and 2015, aged 7 to 27 years old, the students in our study, have different attitudes towards the protection of the environment as mentioned also elsewhere [35]. Bank of America predicts that Gen Z will become “the most disruptive generation ever”. It is reported that no generation before them has shown the same interest in any societal issue. Data shows that Gen Z is the generation that’s most worried about global warming (38% of the population in the U.S.) [36,37,38]. According to these perceptions, also in our study they seem more sensitive about safeness of amalgam for the environment, patient, and staff, overall. The optimistic part confirmed almost everywhere in the relevant literature, is that for this generation, learning is an active experience and no longer relegated to the passive learning approaches of the past [39,40,41,42]. So, also in our case, they asked for more information to be shared during their studies on environmental practices. Current dental students could be pioneers nowadays for the evolvement of the “green dentistry project” if given constant and reliable educational perspectives on “green dental processes”. They could then successfully open the path for more sustainable dental processes for the upcoming Gen A.

Another interesting finding to discuss in our study is the fact that living outside urban centers, seem to influence positively the sensitivity of all participants, students, and dentists, about the safe use and disposal of amalgam. People living in small communities under 5000 people, outside urban centers, are more dedicated in taking relevant safety measures for both patients and staff as also mentioned elsewhere [34]. This could possibly be attributed to the more direct relationship they have to the environment and its needs.

Finally, about the concern of patients towards the use of amalgam, the opinions of both students and dentists were not influenced by a single variable but by all general characteristics of each group. In relevant research involving Turkish dentists though, participants’ use and views on dental amalgam in relation to gender, field, experience, and title showed that most dentists working in a private practice, from 0 to 5 years did not use dental amalgam (60%), mainly at the request of their patients. The clinical cases where dental amalgam was used were mainly in large restorations or in patients with poor oral hygiene. In that research, most respondents reported that amalgam is safe for both the physician and the patient while in smaller percentages it was considered inappropriate [26]. These results are in part common with our findings despite the differentiation in the profile of the respondents. However, the finding that gender could influence statistical differences on amalgam use [26], was not proven in our study. It is noteworthy that dentists continue to use dental amalgam in other studies too although they believe that it has negative effects on their health especially because of the potential toxicity of composite resins to human health and the environment too [37,38].

It seems that information sharing and engagement in environmental actions through “green dentistry approaches” will make dental students and professionals to adapt and be more flexible in a world that changes rapidly, to have a sustainable modern practice. The lack of public and professional awareness is the greatest driver to engage with a positive change in behavior and attitudes. Awareness through education is key at all levels and this should be the bedrock of future strategies [41]. Although there is increasing awareness of the problems associated with global warming, there exist a lack of knowledge on how to become more environmentally sustainable almost in all fields of human activities [42,43]. Thus, eco-friendly dentistry is more and more coming up as an issue of systemic dental management that oversees holistically the dentist in his interior and exterior environment to promote health for patients, himself and nature. By this sense, eco-friendly dentistry tries to promote the dental life expectancy for all people involved by insisting to reduce waste and pollution, save energy, water, and money and to incorporate high tech innovations, focus on wellness, and integrative practices [1,27,28,44,45,46]. Under this scope, attempts should continue to reduce chemicals’ detrimental impact on the environment and promote environmental awareness and sustainability to patients in the dental field. These attempts will encourage sustainable practices by reducing further resource consumption, waste and safe disposal of materials containing hazardous metals such as Hg.

Despite the serious questions arising all over the world about ecologically friendly professional approaches, after COVID-19, dental professionals, should use a systemic management tool that could be developed by governments and professionals’ associations and should be based on the 4As approach [44]: (a) asking the right questions concerning eco-friendly practicing (why do we need it, what is our motivation to certain actions, what needs will be considered personally and professionally in order to make sustainable decisions) and gathering all the details from the professionals on their routine dental practices, as we tried to do in this study; (b) assessing the measures already taken and what all practices can modify not because of the legislation but from a systemic point of view, thus considering “self” as a whole with the environment; (c) advising those professionals currently in action with a clear set of guidelines and economical support on new “green” equipment (dental equipment renovation is expensive for middle class practices to be constantly auto-supported for “green” changes); (d) assisting them in green actions within their dental office and the community with information material, target-specific online seminars, literature reviews and live environmental actions.

## 5. Limitations and Future Research Considerations

Limitations of the present study were the difference in the number of participants in the two groups and the fact that dentists in the second group were those attending continuing education seminars, meaning they were those that care more for legislation or environmental issues anyway. Of course, despite this fact, the group’s attitudes prove the need for further information on the research issue. Thus, current data could be used for future screening of environmental thermometer and engagement acts for both students and dental professionals.

## 6. Conclusions

The present study has shown that professionals and students consider that amalgam brings a negative burden on the environment to a small or moderate extent, which is in line with most studies worldwide. They do not propose or use it any more for posterior conservative restorations. Undergraduate education, years in the profession and practicing in non-urban regions play an important role in their sensitivity about safety and environmental accountability issues. Dental students and professionals support lifelong learning through seminars and literature review on environmental approaches. Overall, the use of dental amalgam is currently very limited. Dental modern practices should encourage information sharing about environmental issues to promote safety and sustainability for both the environment, the patients, and the professionals.

## Figures and Tables

**Table 1:** Significant values for dentists.

**To what extent do you think that dental amalgam causes environmental harm**							
**Independent Variables**	**At all** **N1 (%)**	**small** **N1 (%)**	**Medium** **N1 (%)**	**Large** **N1 (%)**	**Total**	**X^2^**	***p***
Total	25 (5.4)	153 (33.1)	184 (39.8)	100 (21.6)	462		
**Years of practice**						15.452	0.079
≤5	6 (5.8)	26 (25.2)	51 (49.5)	20 (19.4)	103		
6–15	6 (4.5)	54 (40.9)	46 (34.8)	26 (19.7)	132		
16–25	4 (3.3)	46 (37.7)	42 (34.4)	30 (24.6)	122		
>25	9 (8.6)	27 (25.7)	45 (42.9)	24 (22.9)	105		
**Do you have the necessary equipment for the environmentally safe way of placing and removing dental amalgam?**							
**Independent variables**	**Partly** **N1 (%)**	**Yes** **N1 (%)**	**No** **N1 (%)**	**Total**	**X^2^**	***p***	
Total	176 (38.1)	158 (34.2)	128 (27.7)	462			
**Field of engagement**					20.341	0.061	
Resonstructive Dentistry	3 (42.9)	3 (42.9)	1 (14.3)	7			
General Dentistry	162 (40.2)	129 (32)	112 (27.8)	403			
Pedodontics, Orthodontics	1 (16.7)	4 (66.7)	1 (16.7)	6			
Endodontics	6 (45.2)	2 (15.4)	5 (38.5)	13			
Periodontology	2 (17.7)	6 (50)	4 (33.3)	12			
Prosthodontics, Implantology	1 (10)	7 (70)	2 (20)	10			
Oral Surgery	1 (9.1)	7 (63.6)	3 (27.3)	11			
**To what extent do you think that amalgam restorations can negativelly affect patient’s health?**							
**Independent variables**	**Not at all** **N (%)**	**Small** **N (%)**	**Medium** **N (%)**	**Large N (%)**	**Total**	**X^2^**	***p***
Total	142 (30.7)	183 (39.6)	114 (24.7)	23 (5)	462		
>25	32 (30.5)	45 (42.9)	23 (21.9)	5 (4.8)	105		
**Place of practice**						26.886	0.001
Outside the urban center	26 (21)	46 (37.1)	47 (37.9)	5 (4)	124		
Overseas	4 (50)	2 (25)	0 (0)	2 (25)	8		
Urban Center	112 (33.9)	135 (40.9)	67 (20.3)	16 (4.8)	330		
**To what extent do you think that amalgam restorations can negatively affect the health of dental staff?**							
**Independent variables**	**Not at all** **N (%)**	**Small** **N (%)**	**Medium** **N (%)**	**Large** **N (%)**	**Total**	**X^2^**	***p***
Total	91 (19.7)	178 (38.5)	152 (32.9)	41 (8.9)	462		
**Place of practice**						7.448	0.001
Outside the urban center	23 (18.5)	44 (35.5)	45 (36.3)	12 (9.7)	124		
Abroad	3 (37.5)	3 (37.5)	0 (0)	2 (25)	8		
Urban center	65 (19.7)	131 (39.7)	107 (32.4)	27 (8.2)	330		

**Table 2:** Significant values for students.

**To what extent do you think that dental amalgam causes environmental harm?**								
**Independent variables**	**Not al all** **N (%)**	**Very small** **N (%)**	**Small** **N (%)**	**Medium** **N (%)**	**Big** **N (%)**	**Total**	**X^2^**	***p***
Total	1 (1)	9 (9)	21 (21.2)	53 (53.5)	15 (15.3)	99		
**Year of study**							9.26	0.05
4°	1 (6.3)	2 (12.5)	1 (6.3)	11 (68.8)	1 (6.3)	16		
5°	0 (0)	7 (8.4)	20 (24.1)	42 (50.6)	14 (16.9)	83		
**To what extent do you think that amalgam can negatively affect the health of dental staff?**								
**Independent variables**	**Not at all** **N (%)**	**Very small N (%)**	**Small** **N (%)**	**Medium** **N (%)**	**Big** **N (%)**	**Total**	**X^2^**	***p***
Total	8 (8)	27 (27.3)	25 (25.3)	36 (36.4)	3 (3)	99		
**Place of practice**								
Outside urban centers	0 (0)	6 (42.9)	1 (7.1)	6 (42.9)	1 (7.9)	14	13.603	0.09
Practice abroad	0 (0)	0 (0)	3 (50)	2 (33.3)	1 (16.7)	6		
Urban centers	8 (10.5)	20 (26.3)	20 (26.3)	27 (35.5)	1 (1.3)	76		
**To what extent do you think that amalgam restorations can negativelly affect patient’s health?**								
**Independent variables**	**Not at all N (%)**	**Small** **N (%)**	**Medium** **N (%)**	**Big** **N (%)**	**Total**	**X^2^**	***p***	
Total	19 (19.2)	34 (34.3)	20 (20.2)	26 (26.3)	99			
**Field of engagement**						28.209	0.05	
Reconstructive Dentistry	3 (42.9)	2 (28.6)	2 (28.6)	0 (0)	7			
General dentistry	12 (21.8)	17 (30.9)	14 (25.5)	12 (21.8)	55			
Pedodontics, Orthodontics	2 (16.7)	4 (33.3)	2 (16.7)	4 (33.3)	12			
Endodontics	0 (0)	3 (100)	0 (0)	0 (0)	3			
Periodontology	0 (0)	4 (80)	0 (0)	1 (20)	5			
Prosthodontics, Implantology	2 (25)	2 (25)	1 (12.5)	3 (37.5)	8			
Oral Surgery	0 (0)	1 (16.7)	0 (0)	5 (83.3)	6			

## Data Availability

Data available upon request.
